# A comparison of neonatal outcomes after taking progesterone pills and progesterone intramuscular injections in preterm labor: An RCT

**DOI:** 10.18502/ijrm.v21i7.13888

**Published:** 2023-08-23

**Authors:** Maryam Dalili, Moeeneh Barkhori-Mehni, Fatemeh Karami Robati

**Affiliations:** ^1^Department of Obstetrics and Gynecology, School of Medicine, Kerman University of Medical Sciences, Kerman, Iran.; ^2^Maternal-Metal Medicine Research Center, Shiraz University of Medical Sciences, Shiraz, Iran.; ^3^Clinical Research Development Unit, Afzalipour Hospital, Kerman University of Medical Sciences, Kerman, Iran.

**Keywords:** Obstetric labor, Premature, Progesterone, Oral, Injections.

## Abstract

**Background:**

Approximately two-thirds of infant mortality within the first year of life are caused by preterm labor (PL).

**Objective:**

This study aimed to investigate the effects of progesterone-based compounds to prevent PL.

**Materials and Methods:**

This randomized clinical trial study was conducted on 146 pregnant women admitted to Department of Obstetrics and Gynecology, Afzalipour hospital in Kerman University of Medical Sciences, Kerman, Iran in June 2019. The participants with PL received Tocolytic and 12 mg Betamethasone in 2 doses over 2 days to mature the fetus's lungs. Stopping PL was considered a 12-hr period without any contractions after finishing the Tocolytic. Following the successful cessation of PL, the participants were monitored for 48 hr. Subsequently, the participants were divided into 2 groups. Participants received 200 mg Lutogel capsules orally per day in group A while group B received a weekly dose of 250 mg Proluton in the form of intramuscular injection, respectively. Treatment in groups continued until the 36 ${}^{\text{th}}$ wk of delivery. The participants were followed-up weekly, and if any signs of PL were detected, an obstetrician carried out a vaginal examination.

**Results:**

The incidence of PL was the same in both groups. There was no significant difference in the latent phase, average birth weight, and the neonatal intensive care unit admission frequency (p = 0.07, 0.17, 0.58, respectively) between groups.

**Conclusion:**

No difference in the results obtained from theneonataloutcomes evaluated in groups. Both medications similarly led to recovering pregnancy and neonatal outcomes caused by PL. Applying the oral form with similar beneficial effects were pointed out in this study, which can be a solution to the issues caused by numerous injections that are inevitable in the injected administration of this medicine.

## 1. Introduction 

Preterm labor (PL) is one of the most crucial medical topics that attracts the attention of obstetricians and pediatricians. PL is the beginning of labor pain after 24 wk and before 37 wk of the gestational age (1). Two-thirds of death cases in the first year of life are caused by PL. This medical condition leads to prolonged neonatal intensive care unit (NICU) admission, increased hospitalization costs, and imposes extra charges upon participants, their families, and the healthcare system. It may cause serious physical and mental issues during infancy and older ages (2).

Respiratory distress syndrome and intracranial hemorrhage are short-term complications, while cerebral palsy, epilepsy, brain retardation, blindness and deafness, and permanent neurogenic issues are considered some of the long-term complications in premature infants. Such problems not only put a lot of psychological pressure on parents and physicians, but also impose a lot of economic and social burden on any society (3). Etiologically, PL is multifactorial, and its causes are unknown in most cases (4). Some of the possible causes are Chorioamnionitis, urinary and reproductive tract infections, anatomic uterine and cervical defects, fetal defects, placental abnormalities, multiple pregnancies, mother's medical conditions and surgeries during pregnancy, and socioeconomic conditions (5).

Despite all the complications and issues caused by PL, this condition is considered the top preventable prenatal cause of death. The most important factor to determine as an infantile complication in PL is the maturity level of the lungs, which is in close relation with maternal and fetal complications. Tocolytic medicines such as beta-agonists, prostaglandin inhibitors, nifedipine, and magnesium sulfate are applied to cure the acute phase of PL, to delay the delivery, and to achieve the effects of corticosteroids on the fetus's lungs (6).

Progesterone was given attention as a preventive factor of PL in the early 1960s. Progesterone increases in the mother's plasma during pregnancy (7). That is why applying progesterone to stabilize and relax uterine conditions has been subject to various investigations (8). Regarding the contradictory results concerning the application of progesterone to prevent PL, some meta-analyses have been carried out on the published research (9). All the researchers obtained evidence that proves the benefits of progesterone in decreasing the rate of PL to some degree. However, all these researchers point out the necessity of further studies in this regard (10).

This study compared pregnancy and neonatal outcomes after taking progesterone pills and intramuscular progesterone injections in PL.

## 2. Materials and Methods 

### Study design and sample collection

This randomized clinical trial study was conducted on 146 pregnant women admitted to Department of Obstetrics and Gynecology at Kerman University of Medical Sciences, Kerman, Iran in June 2019.

The inclusion criteria were: the women who were admitted for the PL treatment, single pregnancy with a live fetus, healthy amniotic sac, gestational age between 24-36 wk confirmed by last menstrual period and ultrasound of the first 3 months of pregnancy, lack of cerclage, labor pains in the form of contractions alternating 2 times in 10 min or 8 times in 60 min and cervical dilatation ≥ 1 cm. (Dilatation: means that the cervix opens. As labor nears, the cervix may start to thin or stretch (efface) and open (dilate). This prepares the cervix for the baby to pass through the birth canal (vagina).

The exclusion criteria for this study were preterm premature rupture of membranes, vaginal bleeding, cervical dilation > 3 cm, fetal death or fetal distress, maternal systemic diseases, known uterine anomalies (with a history or ultrasound), history of taking any medication other than the usual supplements during pregnancy, polyhydramnios and oligohydramnios, fetal anomaly, suspicion of intrauterine infection due to clinical signs of mother and fetal heart rate, being dangerous for the mother to continue the pregnancy due to medical reasons, and lack of cooperation of mothers during the study.

The estimated sample size was calculated by 2-sample comparison of proportions (p1 = 0.1250, p2 = 0.0000, power = 0.8000, alpha = 0.0500) according to previous study (11).

### Data collection procedures

Initially, demographic information including gestation age, time of arrival at the hospital, gravidity, parity, gestation age according to last menstrual period and early pregnancy sonography, onset time of labor pain, and the participant's medical history were recorded. Then participants' clinical indications including the number of uterine contractions per hour and the duration of each contraction which was carried out using tokometry, and vaginal examination with the consent of the patient to determine the amount of dilatation at the arrival time were recorded.

In the next step, those eligible to enter the study were selected for further intervention and examinations. The participants with PL received tocolytic depending on the situation to control the acute phase of PL. In order to mature the fetus's lungs, the participants were given 12 mg Betamethasone in 2 doses over 2 days. Stopping PL was considered as a 12-hr period without any contractions after finishing the tocolytic. Participants were monitored for 48 hr until the acute phase and stopping PL were under control. After stopping labor pain successfully, the participants were divided into 2 groups (n = 73/each) according to a random number table. The patient and physician were aware of the type of treatment, and only the person evaluating the data was unaware of the type of treatment at the end. The statistical consultant performed randomization blindly and provided the results to the researcher, and the researcher assigned the participants to the intervention groups.

In group A, participants received 200 mg Lutogel capsules (each capsule containing 200 mg of Utrogestan natural micronized progesterone) orally per day. In group B, participants received a weekly dose of 250 mg of Proluton in the form of intramuscular injection. Treatment in both groups continued until the 36 ${}^{\text{th}}$ wk or delivery. The participants were required not to take any other tocolytic agents or medicines without the therapist's permission in charge. All the participants were followed up weekly in the women's clinic, and after diagnosing any symptoms of PL, a vaginal examination was carried out by an obstetrician. If the delivery process was confirmed, the mother was observed in the hospital for further examinations and received supportive measures.

Primary neonatal outcomes (birth weight, gestational age at delivery, and the number of hospitalized neonates in each group) were evaluated after delivery based on patient documents. The latency period was assessed from the beginning of the intervention until delivery based on patient documents. No secondary neonatal outcomes were observed.

After the delivery, the conditions of birth were evaluated based on the recorded time of the delivery, delivery method, and mentioning the reason in case of carrying out a cesarean in addition to child weight estimate, Apgar score, the necessity of hospitalization in NICU, and hospitalization length. Then latent period length (length of time spent from the beginning of the intervention to the delivery) was calculated.

(Apgar score: the Apgar score is a test given to newborns soon after birth. This test checks a baby's heart rate, muscle tone, and other signs to see if extra medical or emergency care is needed. Babies usually get the test twice: 1 min after birth and again 5 min after they are born).

Effacement means that the cervix stretches and gets thinner. In midwifery, 2 fingers mean 2 cm.

### Ethical considerations

Helsinki principles are declared in this study. The Ethics Committee of Kerman University of Medical Sciences, Kerman, Iran approved this study (Code: IR.KMU.AH.REC.1397.155). In addition, this study was approved by the Iranian Registry of Clinical Trials (IRCT) on 05-04-2020. Before starting the clinical trial, all participants were informed of the intervention methods, and written informed consent was obtained from each patient.

### Statistical analysis

Descriptive (frequency, percentile, mean, and standard deviation), analytical (Chi-square test) methods and Statistical Package for the Social Sciences, version 22.0, SPSS Inc, Chicago, Illinois, USA (SPSS) were used to analyze the data. P < 0.05 was considered as significant.

## 3. Results

**Figure 1:**
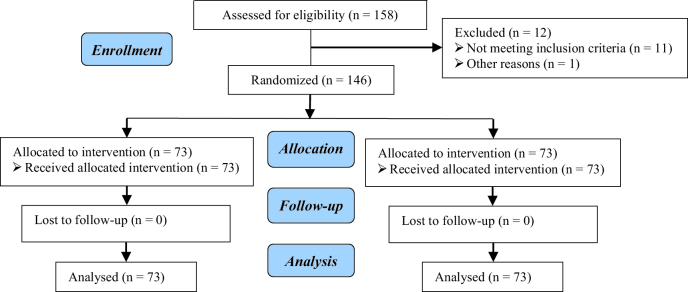
CONSORT flow diagram.

This study was performed on 146 pregnant women admitted to the Department of Obstetrics and Gynecology in 2019. In total, 158 cases were assessed for eligibility, of which 12 cases were excluded (11 cases did not meet inclusion criteria, 1 case declined to participate, and one other reason) (Figure 1).

The mean age of the participants was 28.75 yr (17-42 yr). The mean age of the participants in the groups A and B were 29.5 and 28.0 yr, respectively, without a significant difference (p = 0.12). Most of the participants were in the group of 18-35 yr. The mean age of gestation in the groups A and B was 31.7 and 31.5 wk, respectively.

No difference was observed between the medication-start age groups (p = 0.57). The highest number of women with PL was in the group of 30-34 wk. Most participants were categorized as 2 fingers or less dilation in both groups and less than 20% effacement. No difference was observed between groups in terms of dilatation and effacement.

The average gestation age at the time of birth was 36.7 and 36.3 wk in groups A and B, respectively (p = 0.18). The length of the latent phase was 4.5 wk in the group A and 5.2 wk in the group B (p = 0.07). The highest number of deliveries in both groups was a vaginal delivery. No difference was observed between the 2 groups according to delivery methods (p = 0.86) (Table I). 52.1% and 63% of infants in the groups A and B, respectively had weights higher than 2500 gr (p = 0.18) (Table II).

**Table 1:** Demographic characteristics of participants (n = 146)


**Characteristics**		**Group A**	**Group B**	**P-value**
**Mother's age (yr)**				
	**< 18**	0	2 (3)	
	**18-35**	61 (83.6)	56 (76.7)	
	**> 35**	12 (16.4)	15 (20.5)	0.12
**Employment status**				
	**Employed**	32 (43.83)	30 (41.09)		
	**Housewife**	41 (56.16)	43 (58.9)	0.74
**Smoking status**				
	**Negative**	71 (97.26)	70 (95.89)	
	**Positive**	2 (2.73)	3 (4.1)	0.65
**Primipara parity**		29 (39.72)	26 (35.61)		0.61
**Delivery method**				
	**Cesarean**	30 (41.09)	28 (38.35)	
	**Vaginal**	43 (58.9)	45 (61.64)	0.86
**The first cesarean compared to all cesareans**		11 (37.5)	10 (35.7)		0.81
**Intervention start age (wk)**				
	**24-30**	12 (16)	14 (19.2)	
	**30-34**	48 (65.5)	40 (54.8)	
	**34-36**	13 (17.8)	19 (26)	0.57
**Cervical dilation**				
	**< 2f**	58 (79.7)	57 (77.41)		
	**2f-3 cm**	15 (20.3)	16 (22.59)	0.21
**Effacement**				
	**20%**	49 (67.2)	47 (64)	
	**30%**	24 (32.8)	26 (36)	0.19
**Birth age (wk)**				
	**≥ 37 **	48 (65.45)	34 (46.7)	
	**34-37**	20 (27.39)	33 (45.2)	
	**< 34 **	5 (7.16)	6 (8.21)	0.059
**First cesarean section**		26 (35.7)	27 (37.5)		0.81
Data presented as n (%). Chi-square test				

**Table 2:** Infants' outcomes


**Variable**		**Group A**	**Group B**	**P-value**
Weight (gr)*				
	**< 2500**	35 (47.9)	27 (37)	
	**≥ 2500**	38 (52.1)	46 (63)	0.18
Apgar score**				
	**Minute 1 ≥ 7**	72 (98.6)	70 (95.9)	
	**Minute 1 < 7**	1 (1.4)	3 (4.1)	0.62
	**Minute 5 ≥ 7**	72 (98.6)	71 (97.3)		
	**Minute 5 < 7**	1 (1.4)	2 (2.7)	1.00
NICU admission*				
	**Need for NICU admission**	22 (30.1)	19 (26)	
	**No need for NICU admission**	51 (69.9)	54 (74)	0.58
	**Need for NICU admission 24 hr < in** **relation to all deliveries**	10 (13.7)	12 (16.4)		
	**No need for NICU admission 24 hr < in** **relation to all deliveries**	63 (86.3)	61 (83.6)	0.64
Data presented as n (%). *Chi-square and, **Fisher-exact test, NICU: Neonatal intensive care unit				

## 4. Discussion

In this clinical trial, the influence of progesterone administration (oral/injection) as a medication with 2 goals were investigated; one of which was prolonging pregnancy after occurring PL, and the other one was examining its effects on prenatal outcomes. The conclusion of this research is as follows: first, both medications have a beneficial impact on prenatal health, and second, both methods are equally effective in prolonging pregnancy and delaying delivery.

In general, this research indicates that the delivery rate above 37 wk was 53.3% in the whole population and 46.2% and 64.4% for groups B and A, respectively. No significant difference was observed between the 2 groups regarding the delivery method. In this research, the percentage of cesarean sections in groups B and A was largely equal with 38% and 40% successively.

The average weight of newborn infants was 2989 gr. The mean weight was 2759 and 2619 gr in the groups B and A, respectively (p = 0.17).

28% of infants were hospitalized in the NICU (30% in group A and 26% in group B). 16% of infants required hospitalization longer than 24 hr (17% in group B and 15% in group A). No significant difference was observed between the 2 groups.

3 studies were performed on oral progesterone versus placebo (including 386 participants: 196 on oral progesterone and 190 on placebo). The results of the meta-analysis revealed the risk of preterm delivery (PTD) at 37 wk of gestation (42% vs. 63%; p = 0.0005). Premature delivery at 34 wk of pregnancy (29% vs. 53% p < 0.00001) was lower in the oral progesterone group than in the placebo group. Infant mortality was also found to be significantly lower (5% vs. 17%; p = 0.001). The NICU admission was lower in the oral progesterone group. Infants' weight was higher in the progesterone group. Congenital side effects with oral progesterone included dizziness, drowsiness, and vaginal dryness, and no serious side effects have been reported. In this meta-analysis, oral progesterone effectively prevented preterm birth (PTB) and reduced mortality during pregnancy in asymptomatic pregnancies with a history of spontaneous PTD compared with placebo. Side effects with oral progesterone also increased compared with placebo, but none were serious. Although no meta-analysis was performed to compare the 2 different forms of progesterone, all studies showed that progesterone was used in treating PL and that no serious side effects occurred following progesterone use (12), which was consistent with our study. However, the present study was performed on women with PL without considering the previous labor history, and therapeutic intervention was performed in both groups.

A study in Egypt showed that although both forms of progesterone improve pregnancy outcomes, the vaginal form has been more effective. In this study, the mean age of delivery was 36 ± 5.7 wk, and the mean gestational age was $36\frac{4}{7}$ wk in the oral group and $37\frac{1}{7}$ wk in the vaginal type of progesterone (p = 0.012). Also, the weight of neonates in the vaginal progesterone group was 3 kg, which in contrast to 2.87 kg in the oral drug group, showed significantly higher importance (p < 0.001) (11). These results differ from the present study results, which showed the same effect of taking both oral and injectable forms of progesterone. However, in our study, oral progesterone was compared with the injectable form, which may be one of the reasons for the difference in the results of these 2 studies used as different progesterone compounds. However, according to all these studies, the overall neonatal outcome in all groups of progesterone users is good in various forms. No progesterone-related neonatal complications were seen in any of the studies.

In one study on 212 pregnant women, a primary outcome was the prevalence of spontaneous PTD. Secondary outcomes included gestational age at birth and hospitalization in the NICU. In the progesterone group, the need for weeks of medication was higher (35.4 vs. 33.9 wk). The 2 groups had similar frequencies of delivery methods and postpartum complications. In the progesterone group, the infant mortality rate was lower (3.7 vs. 25.2%). In addition, in the progesterone group, the infant admission rate in the intensive care unit was reduced. Finally, oral progesterone was effective in preventing PL and was worth researching in the future (12). Our study aimed to evaluate the effect of oral progesterone compounds compared to the injectable form. The results show that oral progesterone compounds do not have a weaker effect on the injectable form and are equally effective in preventing PTD.

In another study, in the oral progesterone group, the duration of the latent phase was significantly longer (29.39 ± 16.21 vs. 23.07 ± 23.47 days, p = 0.014). The number of preterm infants was significantly lower in the oral progesterone group (33% vs. 58%, p = 0.034). The mean weight of neonates in the group treated with oral progesterone was higher (2.64 ± 0.58 vs. 2.47 ± 0.44 kg, p = 0.009). As a result of the study, it was reported that oral progesterone is effective in increasing the length of pregnancy and reducing neonatal mortality and morbidity (13). This result was consistent with the results of the present study.

In a double-blind, randomized trial study, PTB occurred in 29 participants in the oral progesterone group (39.2%) and 44 participants in the placebo group (59.5%). The mean gestational age at delivery in the oral progesterone group was 36 $\frac{1}{7}$ compared to 34 wk in the placebo group and above (p < 0.001). Birth weight and Apgar score were significantly higher in the progesterone group (14). These results were found to be similar to our study.

In one study, the mean gestational age at delivery (36.3 vs. 36.5) and the rate of PTD at 37 wk (40.4% vs. 48.7%), 35 wk (16.7% vs. 16.8%), and 32 wk (1.5% vs. 0.5%) were found to be similar in the 2 groups (p > 0.05) (15). The results of this study were similar to our research in increasing the latent phase length.

One study aimed to investigate the effect of using vaginal progesterone compounds on prolonging pregnancy after PTD. In this study, 140 pregnant women were divided into 2 groups of 70 after successful cessation of PL pain. In the intervention group, participants were given a 400 mg dose of vaginal progesterone every night, and in the other group, a placebo was started. The mean latency phase until delivery was 36.3 days in the intervention group and 24.22 days in the control group; respiratory distress syndrome 4 (10.8%) was lower than 12 (36.4%) in the intervention group (p = 0.021). The average birth weight in the neonates of the intervention group was 3101 gr and in the control group was 2609 gr, moreover, a significant difference was observed between the 2 groups (p = 0.002). Admission to the intensive care unit (24.3% vs. 39.4%) (p = 0.205); and neonatal sepsis (5.4% vs. 18.2%) (p = 0.136) were reported for progesterone and control groups, respectively. No significant difference was observed in neonatal sepsis and hospitalization in the intensive care unit between the intervention and control groups (16). The results of this study were evidence of the success of progesterone in prolonging pregnancy after PTD, which was consistent with our study. In addition, in this article, no improvement was observed in neonatal outcomes following the use of progesterone. This was contrary to previous studies and our study. Although there was no control group in our study, in both intervention groups, our study had a good neonatal outcome.

Given that PTD is the leading cause of neonatal mortality and long-term disability, 2 reported clinical trials reported lower rates of PTD using 17 alpha hydroxyprogesterone caproate and a progesterone vaginal suppository. However, it is unclear whether high-risk women benefit from this treatment or whether social, cultural, racial, and genetic differences cause differences in patient response to progesterone. At the end of the article, further research is recommended to identify the at-risk group, optimal gestational age at onset, how to use progesterone dose, and long-term safety (17). The present study also emphasizes the importance of further studies to understand the effect of progesterone compounds to identify all aspects of treatment with these drugs.

## 5. Conclusion

According to this study, it can be conceived that both oral and injected forms of progesterone have the same influence on prolonging pregnancy after successfully stopping delivery. In both groups, a higher percentage of participants underwent an acceptable length of pregnancy and the neonatal outcomes and disabilities caused by PL have been equally reduced to a great extent in both groups, and most infants underwent satisfactory birth effects. In this research, the oral form of progesterone was compared with its injected form, while in today's midwifery; the injected form has found its place and is increasingly used. Its effects have proved how beneficial this medicine is to cure PL and has caused satisfaction for pregnant mothers who formerly had PL and physicians. However, numerous injections and complications caused by them impose issues upon pregnant women. Applying the oral form with similar beneficial effects were pointed out in this study, which can be a solution to the issues caused by numerous injections that are inevitable in the injected administration of this medicine.

##  Conflict of Interest

The authors declare that there is no conflict of interest.

## References

[B1] Mastantuoni E, Saccone G, Gragnano E, Spiezio Sardo AD, Zullo F, Locci M, et al (2021). Cervical pessary in singleton gestations with arrested preterm labor: A randomized clinical trial. Am J Obstet Gynecol MFM.

[B2] Mol BW, Wood S, Rode L, Tabor A, Aboulghar MM, Porcher R, et al (2021). Evaluating progestogens for preventing preterm birth international collaborative (EPPPIC): Meta-analysis of individual participant data from randomised controlled trials. Obstet Gynecol Survey.

[B3] Mathew S, Kumar A (2018). A prospective analysis of the risk factors and the perinatal outcome of preterm labour. Int J Reprod Contracept Obstet Gynecol.

[B4] Jarde A, Lutsiv O, Beyene J, McDonald SD (2019). Vaginal progesterone, oral progesterone, 17‐OHPC, cerclage, and pessary for preventing preterm birth in at‐risk singleton pregnancies: An updated systematic review and network meta‐analysis. BJOG.

[B5] Stewart LA, Simmonds M, Duley L, Llewellyn A, Sharif S, Walker RA, et al (2021). Evaluating progestogens for preventing preterm birth international collaborative (EPPPIC): Meta-analysis of individual participant data from randomised controlled trials. Lancet.

[B6] Zierden HC, Ortiz JI, DeLong K, Yu J, Li G, Dimitrion P, et al (2021). Enhanced drug delivery to the reproductive tract using nanomedicine reveals therapeutic options for prevention of preterm birth. Sci Transl Med.

[B7] Coomarasamy A, Devall AJ, Cheed V, Harb H, Middleton LJ, Gallos ID, et al (2019). A randomized trial of progesterone in women with bleeding in early pregnancy. N Engl J Med.

[B8] Devine K, Richter KS, Widra EA, McKeeby JL (2018). Vitrified blastocyst transfer cycles with the use of only vaginal progesterone replacement with endometrin have inferior ongoing pregnancy rates: Results from the planned interim analysis of a three-arm randomized controlled noninferiority trial. Fertil Steril.

[B9] Jarde A, Lutsiv O, Park CK, Beyene J, Dodd JM, Barrett J, et al (2017). Effectiveness of progesterone, cerclage and pessary for preventing preterm birth in singleton pregnancies: A systematic review and network meta‐analysis. BJOG.

[B10] Wood SL, Williams BN, Szychowski JM, Owen J (2020). The effect of intramuscular 17α-hydroxyprogesterone in women screened for shortened cervical length. Am J Perinatol.

[B11] Abdelaziz MH (2017). Oral versus vaginal progesterone in preterm labor. Evid Based Women's Health J.

[B12] Ashoush S, El‐Kady O, Al‐Hawwary G, Othman A (2017). The value of oral micronized progesterone in the prevention of recurrent spontaneous preterm birth: A randomized controlled trial. Acta Obstet Gynecol Scand.

[B13] Choudhary M, Suneja A, Vaid NB, Guleria K, Faridi MMA (2014). Maintenance tocolysis with oral micronized progesterone for prevention of preterm birth after arrested preterm labor. Int J Gynecol Obstet.

[B14] Rai P, Rajaram S, Goel N, Gopalakrishnan RA, Agarwal R, Mehta S (2009). Oral micronized progesterone for prevention of preterm birth. Int J Gynecol Obstet.

[B15] González-Quintero VH, Istwan NB, Rhea DJ, Smarkusky L, Hoffman MC, Stanziano GJ (2007). Gestational age at initiation of 17-hydroxyprogesterone caproate (17P) and recurrent preterm delivery. J Matern Fetal Neonatal Med.

[B16] Borna S, Sahabi N (2008). Progesterone for maintenance tocolytic therapy after threatened preterm labour: A randomised controlled trial. Aust N Z J Obstet Gynaecol.

[B17] O'Brien JM

